# The Role of Microbiota in Primary Sclerosing Cholangitis and Related Biliary Malignancies

**DOI:** 10.3390/ijms22136975

**Published:** 2021-06-28

**Authors:** Burcin Özdirik, Tobias Müller, Alexander Wree, Frank Tacke, Michael Sigal

**Affiliations:** Department of Hepatology and Gastroenterology, Campus Virchow Klinikum (CVK) and Campus Charité Mitte (CCM), Charité Universitätsmedizin Berlin, Augustenburger Platz 1, 13353 Berlin, Germany; burcin.oezdirik@charite.de (B.Ö.); tobias.mueller@charite.de (T.M.); alexander.wree@charite.de (A.W.); frank.tacke@charite.de (F.T.)

**Keywords:** primary sclerosing cholangitis, gut-liver axis, microbiome, microbiota, cholangiocellular carcinoma

## Abstract

Primary sclerosing cholangitis (PSC) is an immune-related cholangiopathy characterized by biliary inflammation, cholestasis, and multifocal bile duct strictures. It is associated with high rates of progression to end-stage liver disease as well as a significant risk of cholangiocarcinoma (CCA), gallbladder cancer, and colorectal carcinoma. Currently, no effective medical treatment with an impact on the overall survival is available, and liver transplantation is the only curative treatment option. Emerging evidence indicates that gut microbiota is associated with disease pathogenesis. Several studies analyzing fecal and mucosal samples demonstrate a distinct gut microbiome in individuals with PSC compared to healthy controls and individuals with inflammatory bowel disease (IBD) without PSC. Experimental mouse and observational human data suggest that a diverse set of microbial functions may be relevant, including microbial metabolites and bacterial processing of pharmacological agents, bile acids, or dietary compounds, altogether driving the intrahepatic inflammation. Despite critical progress in this field over the past years, further functional characterization of the role of the microbiota in PSC and related malignancies is needed. In this review, we discuss the available data on the role of the gut microbiome and elucidate important insights into underlying pathogenic mechanisms and possible microbe-altering interventions.

## 1. Primary Sclerosing Cholangitis (PSC)—An Overview

Primary sclerosing cholangitis is an immune-related cholangiopathy that is characterized by biliary inflammation, cholestasis, and multifocal bile duct strictures of the intra and/or extra-hepatic biliary tree. It is associated with high rates of progression to liver cirrhosis and end-stage liver disease as well as a significant risk of cholangiocarcinoma (CCA) and gallbladder carcinoma [1,2,3]. PSC is a rare disease with an incidence that ranges from 0 to 1.3 cases per 100,000 persons per year [1,4,5]. However, awareness of this disease has grown in the past years. Due to improved access to noninvasive means of imaging the biliary tree (magnetic resonance cholangiopancreatography (MRCP)), it has become clear that PSC accounts for approximately 10% of all liver transplants performed each year [6,7]. Co-occurrence of inflammatory bowel disease (IBD), most commonly ulcerative colitis (UC), has been reported in up to 80% of patients with PSC, and 2–7.5% of IBD-patients develop PSC [3,6,8,9,10,11,12]. It has even been postulated that PSC-associated colitis represents a distinct IBD entity alongside Crohn’s disease (CD) and UC [13,14]. Emerging data suggest that PSC-IBD has distinct clinical features such as a higher incidence of pancolitis, backwash ileitis, rectal sparing and subclinical inflammation with an increased potential for malignant transformation, exceeding the risk of colorectal cancer of IBD patients without PSC [8,13,15,16].

Currently, no effective medical treatment with impact on overall survival is available. Liver transplantation seems to be the only curative treatment option, but there is also a risk of recurrent PSC after transplantation [1,17,18]. However, the development of better therapeutic agents requires a greater understanding of disease pathogenesis. In this context, the gut microbiota, representing a metabolically highly active human ‘organ’, has recently emerged as an important new player in the pathophysiology of PSC [19,20,21,22,23,24,25,26,27]. Thus, in this review, we discuss the available data on the role of the microbiota in PSC, PSC-IBD and biliary cancer types such as CCA and gall bladder cancer. Moreover, we elucidate important insights into underlying pathophysiologic mechanisms and potential microbe-altering interventions.

## 2. Pathogenesis of PSC and the Role of the Gut-Liver Axis

Many aspects of the pathogenesis of PSC remain unclear. However, it is generally accepted that the interplay between genetic predisposition and environmental factors contributes to the development of the disease and its progression [6,28]. Several genetic risk factors are associated with PSC, but they only contribute to a small fraction of disease susceptibility [2,6,28]. Environmental factors play a pivotal role in the pathogenesis of PSC. Of these, the microbiota has emerged as one of the most important potential environmental players in chronic inflammatory diseases [12,29,30,31].

**Figure 1:**
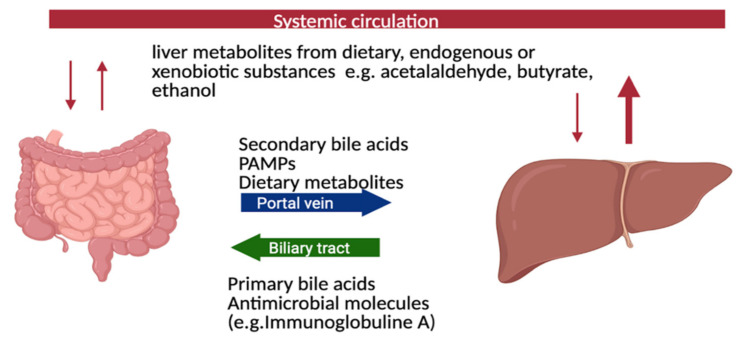
Bidirectional communication between gut and liver through the biliary tract, portal vein, and systemic circulation. The liver communicates with the intestine by transportation of bile salts and antimicrobial molecules (e.g., immunoglobulin A) to the intestinal lumen through the biliary tract, maintaining gut eubiosis by controlling unrestricted bacterial overgrowth. Additionally, endogenous and exogenous metabolites from the liver such as free fatty acids (FFA), ethanol or choline metabolites are transported to the intestine via the capillary system [32,33]. Pathogen-associated molecular patterns (PAMPs).

The close association between PSC and IBD indicates the involvement of the gut-liver axis (Figure 1). Since the gastrointestinal tract harbors a rich and dense microbiota, which is altered in IBD, a potential role of gut microbiota for PSC has been prosed and investigated. Bidirectional communication between the gut and liver is maintained through the biliary tract, the portal vein, and systemic circulation. The liver communicates with the intestine by transportation of bile salts and antimicrobial molecules, such as immunoglobulin A, to the intestinal lumen through the biliary tract, maintaining gut eubiosis by restricting and regulating bacterial growth. Additionally, endogenous and exogenous metabolites from the liver such as free fatty acids (FFA), ethanol or choline metabolites are transported to the intestine via the capillary system [32,33] (Figure 1).

The ‘leaky gut’ hypothesis suggests that leakage of bacterial products across an impaired intestinal epithelial barrier (e.g., damaged by inflammation or infection as well as chemical injury) can lead to translocation of gut microbes or their products (also called pathogen-associated molecular patterns (PAMPs)), which can induce activation of immune cells in the liver [33,34,35]. The activation of Kupffer and hepatic stellate cells and consequent overproduction of proinflammatory cytokines and chemokines, such as tumor necrosis factor, plays a key role in this process and may lead to chronic biliary tract infection and inflammation, resulting in portal fibrosis, and ultimately PSC [32,36,37,38,39]. As a proof-of-principle, Lichtman and colleagues revealed that small intestinal bacterial overgrowth in a rat model, achieved by using a blind jejunal loop, increased translocation of bacterial products and led to cholangiographic changes resembling PSC [40]. Furthermore, analysis of classical markers of bacterial translocation in the serum of 166 PSC patients and 100 healthy controls revealed an elevation in sCD14 and lipopolysaccharide-binding protein (LBP) levels. Strikingly, high LBP levels were associated with poor transplantation-free survival, which indicates that this ongoing gut leakage could have a clinical impact in PSC [41]. In contrast, Björnsson et al. observed no increase in intestinal permeability in PSC patients compared to healthy controls after assessing their differential urinary excretion of lactulose/L-rhamnose [42]. Nevertheless, their study was limited by a small cohort (n = 22 PSC patients).

Further observations in respect to PSC pathogenesis include the homing of gut-derived lymphocytes to the portal areas and the possible presence of an antigenic trigger derived from the colonic content [43,44,45]. Adams and colleagues proposed that the hepatic complications of IBD are mediated by mucosal T cells that are recruited to the portal areas in response to aberrantly expressed mucosal addressin cell-adhesion molecule 1 (MAdCAM1), which is normally restricted to the gut [44]. They revealed that lymphocytes infiltrating to the liver in PSC include T cells recruited to the liver by CCL25 expression, which is a chemokine that activates α_4_β_7_ binding to MAdCAM1 on the hepatic endothelium [43]. Adams and colleagues were the first ones demonstrating in humans that activation of T cells in the gut can be recruited to an extraintestinal site [44]. However, vedolizumab, a α_4_β_7_ integrin antibody that blocks lymphocyte homing towards MAdCAM-1, unfortunately, appears to be ineffective in PSC [46,47].

## 3. Gut Microbiota and PSC Pathogenesis—Mechanistic Insights

The microbiota can be regarded as an environmental factor in itself, being influenced by other environmental factors such as medications, diet and physical activity [48,49,50,51]. Genome-wide association studies have identified 16 risk loci for PSC [52]. Some of these risk loci are associated with microbiome composition e.g., fucosyltransferase 2 (FUT2), which is an important genetic risk factor for disease progression in PSC and host-microbial diversity [53]. Patients with PSC and FUT-2 variants have a different microbial pattern in the bile and colon. Moreover, they are associated with an increased frequency of biliary infections and incidence of dominant stenoses [54]. Another study identified a protective role of the microbiota in pathogenesis of PSC using germ-free mouse models. They demonstrated that germ-free multidrug resistance 2 (mdr2) knockout mice exhibit biochemical and histological features of PSC. Further, they revealed an elevation in cholangiocyte senescence, which is a characteristic of progressive biliary disease [55]. However, the mechanisms remain incompletely explored. One possibility could be the defective bile acid metabolism in germ-free mice, which requires the gut microbiota [55]. Consequently, absence of the anti-inflammatory properties of secondary bile acids might play an important role in increased inflammation and subsequent responses of biliary cells [55]. Further, Nakamoto and colleagues generated gnotobiotic mice by using fecal samples from a patient with PSC and UC harboring *Klebsiella pneumoniae*, which they found to be abundant in patients with PSC [56]. A bacterial-organoid co-culture system demonstrated a damaging effect of *Klebsiella pneumoniae* on epithelial cells. Furthermore, colonization with these bacteria in vivo is associated with bacterial translocation and susceptibility to T helper cell 17-mediated hepatobiliary injuries in mice. Of note, antibiotic treatment targeting *Klebsiella* protected from severe injury. These results highlight a potential role of pathobionts in promoting gut epithelial injury and liver inflammation [56,57].

Furthermore, since PSC is associated with an altered bile metabolism and bile acids can directly affect bacterial survival, such changes could not only impact the colonic microbiota but also influence the upper gastrointestinal microbiota, which then in turn may affect biliary or colonic microbial communities.

These exciting findings give more mechanistic insights into the relationship between microbes and PSC, representing important progress in this field. They do not only reveal a harmful but also a protective effect of gut microbes in respect to pathogenesis. However, while they can serve as a proof-of-principle for potential microbiota-derived mechanisms, findings from animal experiments cannot be directly translated to human PSC populations [51,58]. Thus, in addition, it is important to investigate the relationship between PSC and gut microbiome in human populations.

## 4. Gut Microbiota and PSC—Cross-Sectional Studies

**Figure 2:**
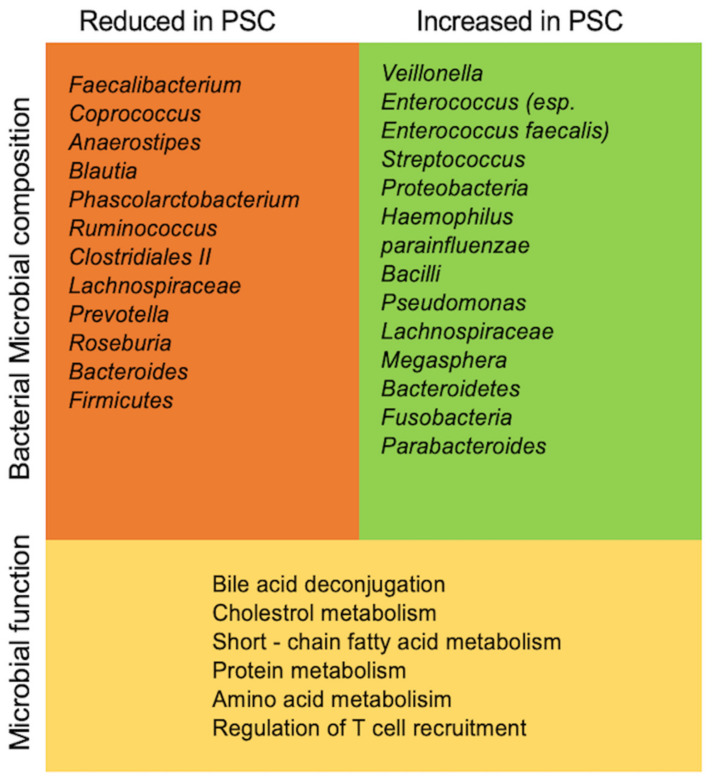
Altered gut microbiota in patients with PSC compared to healthy individuals. Several clinical studies analyzing fecal and mucosal samples have demonstrated that the gut microbiome of individuals with PSC is distinct from that of healthy controls. PSC, primary sclerosing cholangitis.

In the past years, numerous publications aimed to characterize the gut microbiota in PSC/PSC-IBD compared to healthy controls and IBD populations (Table 1). Most of them used 16S rRNA methods to explore the composition of the microbiota. Results of published studies analyzing fecal and mucosal microbiota are summarized in Table 1 and Figure 2.

### 4.1. Fecal Analysis of Gut Microbiota in Patients with PSC

Previous studies of the fecal microbiome in PSC demonstrate that the overall bacterial community is distinct compared to healthy controls without showing any consistency with respect to the specific microbes altered [12,25,27,51,59,60,61,67,68] (Table 1). Sabino et al. analyzed fecal microbiota of 175 individuals (4 cohorts consisting of PSC only, PSC and IBD, IBD, healthy controls), showing that the gut microbiota in PSC (regardless of the presence of IBD) is distinct compared with healthy controls and patients with UC without liver disease. The microbiota of patients with PSC was characterized by decreased bacterial diversity, and a significant overrepresentation of *Enterococcus, Fusobacterium and Lactobacillus* genera. Strikingly, a link between one operational taxonomic unit of the Enterococcus genus and elevated levels of serum alkaline phosphatase (ALP), a marker of disease severity, could be demonstrated [59]. 

Up to now, the abundance of *Veillonella* genus in PSC has been supported by a majority of published reports (Table 1) [25,27,59,60,61]. Nevertheless, this fact is controversially discussed, since the analysis from Sabino et al. revealed that there was no longer a significant association between *Veillonella* genus and PSC when patients with liver cirrhosis were excluded from the analysis [25,59]. *Veillonella* are anaerobic, gram-negative cocci. They contain genes that encode for amine oxidases and are producers of primary amines that could act as vascular adhesion protein-1 (VAP-1) substrates, which is an amine oxidase and adhesion molecule, critical for effector cell recruitment to the liver [25,69,70,71]. Moreover, Veillonella species are associated with other inflammatory and progressive fibrotic conditions like pulmonary cystic fibrosis and idiopathic pulmonary fibrosis and the recurrence of disease in patients with CD undergoing ileocaecal resection [72,73,74]. Of note, in previous studies on CD, Veillonella species alongside *Enterococcus* species were associated with an increased risk of recurrent disease after surgical resection and predisposition to penetrating complications in paediatric patients [74,75]. However, further studies with larger patient cohorts with and without liver cirrhosis, as well as mechanistic studies, are necessary to further understand the role of *Veillonella* species in PSC.

To investigate geographical differences and to overcome single-center bias, Rühlemann and colleagues analyzed the fecal microbiota of a German and Norwegian cohort (*n* = 137). They demonstrated an enrichment of eight taxa in PSC patients compared to healthy controls, independent from the presence of IBD and the use of ursodeoxycholic acid (UDCA) [12]. Compared to healthy controls, many new alterations were identified in PSC such as an increase of Proteobacteria and Parabacteroides, which is a bile-tolerant taxon, associated with changes in cholesterol and bile acid metabolism [76,77]. Furthermore, compared to healthy controls *Bacteroidetes* species were increased and play an important role in protein metabolism as well as in bile acid deconjugation [78,79]. The authors also confirmed previously reported associations for the genera *Veillonella* and *Streptococcus* [27,59].

Bajer and colleagues revealed a marked overrepresentation of *Rothia* genera in PSC, regardless of the presence of IBD [25]. According to the authors and in line with previous reports, the increased abundance of the genus *Rothia,* especially *R. mucilaginosa*, may indicate that oral microbiota is overrepresented in the lower gastrointestinal tract of patients with progressive liver disease [25,31]. Due to the fact that Rothia is sensitive to gastric fluid, the authors assume that a contamination of the intestinal flora by previous endoscopic retrograde cholangiopancreatography (ERCP), particularly with repeated stenting, might be possible [25,80]. In this context, Liwinski et al. investigated oral cavity fluid, duodenal fluid, duodenal mucosa and bile fluid in parallel, revealing that the upper alimentary tract and the bile ducts of PSC patients are likewise affected by microbial dysbiosis. Their study revealed that the bile harbors a unique and diverse microbiome, clearly different from all other communities. They did not reveal any association between previous ERCPs and bile duct composition. However, *Staphylococcus* and *Streptococcus sanguinis* were over-represented in bile samples of patients with PSC who formerly received ERCP [66]. To minimize the risk of contamination of bile fluids during ERCPs the authors additionally performed sequencing of the microbiome from the proximal upper digestive system.

Lemoinne et al. were the first to address fungal changes in the fecal microbiota of patients with PSC and IBD, revealing an increase in biodiversity and an altered composition [61]. They observed an increased proportion of *Exophiala*, a fungi genus of the *Herpotrichiellaceae* family that is mainly involved in infections in immunocompromised hosts [61]. Strikingly, previous case reports already indicated an association between PSC pathology and *Exophiala*. Oztas et al. reported a case of a systemic infection by *Exophiala dermatitidis* mimicking PSC in a young woman without immunodeficiency [81]. Moreover, a case of end-stage liver disease caused by *Exophilia dermatitidis* and characterized by cholestasis and dilatation of intrahepatic bile ducts has been published previously [82]. Furthermore, Lemoinne et al. report a decreased proportion of *Saccharomyces cerevisiae*, which has anti-inflammatory characteristics and was reduced in the microbiota of patients with IBD [61,83]. 

### 4.2. Mucosal Analysis of Gut Microbiota in Patients with PSC

Compared to fecal microbiota analysis, studies of the mucosal microbiome in PSC are less consistent, smaller sample-sized, and fewer in number [51]. Two studies found little to no PSC-specific microbiome alterations, while others showed similar results as fecal analysis [24,26,51,63,65]. In addition, the variability in mucosal microbiota studies may be related to the different sample localizations as well as the different sampling techniques [51]. 

Quraishi’s recently published study is the first to apply an integrative and comparative approach to colonic gene expression, mucosal gut microbiota and immune cell signatures in PSC patients with IBD compared to patients with UC and healthy controls. According to the authors, the microbial alterations and the different gene expression between PSC patients with IBD compared to patients with UC imply a dysregulation of the bile acid metabolism in PSC patients with IBD [65]. Previous studies of biopsies from the colon revealed a significant increase in *Escherichia, Megasphera* and *Lachnospiraceae*, which are able to perform 7α-dehydroxylation, an important step in converting primary to secondary bile acids in the intestine [24,84]. Similar to the *Veillonella* species, these bacteria contain genes that encode for amine oxidases and are all potent producers of primary amines that could act as vascular adhesion protein-1 (VAP-1) [65,69,70,85]. Quraishi et al. demonstrated a reduced abundance of *Prevotella* and *Roseburia*, a butyrate producer. Anaerobic intestinal microbes produce short-chain fatty acids (SCFA) such as acetate, butyrate and propionate by fermenting nondigestible carbohydrates [86,87,88]. Butyrate is a short chain fatty acid, which promotes intestinal barrier function by intracellular tight junction assembly. Therefore, lower butyrate may contribute to weakening of the barrier integrity and dysregulation of the mucosal immunity [65,89]. SCFAs can be metabolized by enterocytes via beta-oxidation, which is thought to maintain an anaerobic environment in the gut—since it is important for survival of anaerobes. Disturbance of this homeostatic process could account for the loss of anaerobes and subsequent loss of SCFAs. SCFAs affect the innate and adaptive immune responses as well as the differentiation of regulatory T cells and disruption of these processes, which may be relevant events for PSC disease processes [90]. In their recently published study, Quraishi and colleagues also found an overrepresentation of *Sphingomonas* genus. These bacteria express amine oxidases and are associated with aberrant homing of gut lymphocytes to the liver, a mechanism that underlies the gut-liver axis [65,91].

Another study included biopsies from the terminal ileum and colon of patients with PSC. The authors demonstrated an increase of the *Barnesiellaceae* family, the *Blautia* genus, and operational taxonomic units (OTU), representing *Clostridiales* [64]. Bacteria from the Clostridiaceae family might play a crucial role in colonic homeostasis since they can affect the number, function and differentiation of colonic regulatory T cells and the production of proinflammatory cytokines [92].

In contrast, Rossen et al. conducted a study, in which they revealed that the colonic mucosa-associated microbiota in PSC is characterized by low diversity and a decreased abundance of *Uncultured Clostridiales II* by using phylogenetic microarrays of ileocecal biopsies [63,93]. Strikingly, in the gut flora of patients with advanced cirrhosis, an increase of *Enterobacteriaceae* and a reduction of *Clostridiales* and *Bacteroides* with a concomitant reduced level of fecal secondary bile acids has been described [94].

In summary, the above-mentioned studies reveal exciting new data on the mucosal and fecal gut microbiota in patients with PSC and represent an important first step in analyzing the association between the gut microbiome and PSC. The reported microbiota profiling studies demonstrate that the gut microbiota in PSC (regardless of the presence of IBD) is distinct compared with healthy controls and patients with IBD without liver disease. Unfortunately, the key question whether PSC is the cause or a result of the microbiota differences remains unanswered. On one hand, the fact that reduced microbial diversity is observed in various inflammatory and metabolic diseases might suggest that reduced microbial diversity is a secondary result of the inflammatory disease [58,95,96,97]. On the other hand, the specific relations between specific bacteria and their impact on the metabolism (e.g., bile acid, protein metabolism (Figure 2)) point towards a leading role of single microorganisms in disease pathogenesis. For instance, *Veillonella*, one of the most reported species enriched in PSC is not only associated with fibrotic conditions but also present in several chronic liver conditions such as primary biliary cholangitis [58,98,99]. However, the importance of the impact of single microorganisms remains unelucidated and will require further investigation. 

While most studies confirm that changes in the microbiota do occur, the qualitative and quantitative changes are highly variable in different studies. The reported broad spectrum of bacteria might be associated with multiple environmental factors such as geographical region, diet, medications, physical activity and hygiene, which can induce substantial shifts in the microbiome composition [48,49,50,51]. One of the main limitations of most of the reported microbiota studies is that the influence of a wide range of environmental factors has not been considered. Thus, it remains unclear, if the significant microbiota alterations in PSC patients are stable even under different environmental conditions (e.g., diet, geographical regions). To further elucidate this question in the context of PSC, Rühlemann et al. performed a multicenter study (Germany and Norway) that included dietary patterns (but only from the German cohort) [12]. Analysis of dietary patterns did not reveal general differences between individuals based on disease status. According to the authors, only minor influences on beta diversity were observed, without association to the clear disease-associated microbial shift [12]. Similarly, studies that analyzed the impact of UDCA treatment revealed no effect on the gut microbiota, since even after exclusion of patients taking UDCA the most common bacterial genera associated with PSC dysbiosis remained [12,25,27,59]. The influence of antibiotic use on the gut microbiota has previously been demonstrated in multiple studies, however, there is no general consensus on how to adjust for antibiotic use in microbiome analysis. While Sabino et al. included all patients who had not taken any antibiotics within the previous month, Bajer et al. used a cutoff of three months and Kummen a cutoff of one year. However, none of the studies observed substantial differences related to antibiotic use. Due to the lack of multicenter studies and studies systematically analyzing the impact of environmental factors (differing diets, medication, physical activity), these questions remain unelucidated, necessitating further investigations to assess the robustness of the observed alterations.

Another important aspect is that studies focusing on fecal analysis reveal consistent results, while results on the mucosal microbiome are highly divergent. Despite studies that report a more accurate microbiome representation by analysis of mucosal samples, review of the studies mentioned above fails to reveal a clear superiority of mucosal analysis [30,51] (Table 1). Thus, more studies comparing the different analysis techniques (fecal vs. mucosal microbiome) in patients with PSC should be performed in the near future.

Although these results represent critical progress in this field, they are also associated with a number of limitations such as small sample size (*n* < 30 on average, *n* < 137 overall), the lack of cross-regional analysis and analysis of additional environmental factors influencing the gut microbiome. Most importantly, functional analyses to further understand the mechanisms underlying the association between gut microbiome and PSC are urgently needed [51]. More studies using metagenomics, proteomics, metatranscriptomics and metabolomics similar to Quraishi’s integrative pilot analysis would enrich this still underexplored field [51,58].

### 4.3. Gut Virome

Although the main focus of gut microbiome research and this review has so far been the bacterial compartment, the gut virome is also present but largely uncharacterized. At present, there are no published studies analyzing the gut virome in PSC patients. Sequencing of the DNA of virus-like particle preparations from fecal samples of IBD patients revealed a significant expansion of Caudovirales bacteriophages and a reduction of Microviridae compared with controls [58,100]. The viromes of patients with CD and UC were disease- and cohort-specific and most importantly, a positive correlation between bacteriophage enrichment and disease activity was shown [58,100]. The authors of the study speculate that the gut virome may contribute to intestinal inflammation by lysis of bacteria during the normal life cycle of a bacteriophage, releasing PAMPs that trigger inflammatory cascades or by induction of a humoral immune response in the host [58,100]. In the context of alcoholic hepatitis, Jiang et al. not only demonstrated that viral taxa are altered in fecal samples from patients, but also revealed an association with disease severity and mortality [101]. However, since there are no studies on the gut virome in PSC, IBD or chronic liver diseases, its potential role in these conditions remains unclear.

## 5. PSC and Occult Viral Infections

The role of occult viral infection in PSC patients is still unelucidated since only a small number of studies and cases with concomitant or previous occult viral infections have been reported.Moreover, current evidence does not reveal consistent or reproducible data to support a role of viruses in ongoing pathogenesis of PSC. Ponsioen et al. analyzed potential agents that increase the risk for PSC. These include bacteria such as Chlamydia spp. as well as multiple virus types such as Epstein-Barr virus, Cytomegalovirus, Mumps, Measles, Coxsackie 1-6 and hepatitis A, B and C. Their results did not reveal evidence of higher titers of the tested viruses amongst PSC patients [102]. Gerorgiadou et al. investigated the presence of occult hepatitis B infection in patients with PSC and failed to detect a significant association [103]. The prevalence of PSC in association with hepatitis C infection is very rare; so far, there are only three reported cases of patients with an initially diagnosed hepatitis C infection who developed PSC during the course of the disease [104,105]. Since larger clinical studies evaluating the role of viral infections in PSC are still lacking, future clinical and experimental studies in this field are urgently required.

## 6. Gut Microbiota as a Therapeutic Target and Future Approach

More insights into the impact of the gut microbiota in PSC and PSC-IBD may lead to the development of useful microbe-altering interventions. In this context, previous studies have already reported evidence that manipulation of the gut microbiota could potentially affect the disease process in PSC. A short overview of therapeutic agents targeting the gut microbiota is provided in Table 2.

### 6.1. Antibiotic Treatment

Emerging data on oral antibiotic treatment (vancomycin, metronidazole, minocycline) revealed a significant improvement in PSC disease activity and progression [107,108,110,112]. In addition to improvement of clinical symptoms, a significant decrease in Mayo-Risk-Score and in serum alkaline phosphatase (ALP) levels could be demonstrated [107,108,112]. The Mayo-Risk-Score is used to predict survival up to 4 years and normalization of ALP is linked to a better long-term outcome and transplant-free survival [113,114]. In particular, vancomycin has evolved as a potential therapeutic agent [107,115]. Its exact mechanisms of action remain to be elucidated but are assumed to be mediated through targeting of the microbiome as well as through anti-inflammatory and possibly immunomodulatory activities. Vancomycin has a relatively narrow antibiotic spectrum and specifically targets gram-positive bacteria such as *Clostridiales*. Since *Clostridiales* biotransforms primary bile acids into secondary bile acids in the distal small intestine and colon, it is possible that the effects of vancomycin on gut microbes influence bile acid metabolism, which in turn might reduce disease activity [112,116,117].

Supporting this notion, a recent RCT including 20 patients with metabolic syndrome revealed not only a reduced fecal microbial diversity with a drop in *Firmicutes* and an increase in *Proteobacteria*, but also a reduction of hydrophobic secondary bile acids after oral vancomycin treatment [112,115]. Furthermore, in a recent mouse study, vancomycin reversed mycophenolate mofetil (MMF)-induced gastrointestinal toxicity by restoring the MMF-induced gut dysbiosis [118]. The mechanisms contributing to MMF-related gastrointestinal toxicity have yet to be fully characterized. However, one leading hypothesis is that MMF changes the gut microbiota composition by selection for β-glucuronidase-expressing bacteria. An up-regulation of β-glucuronidase activity in the gut induces chronic inflammation and weight loss [118]. In addition, a study in 14 PSC UC children demonstrated an increase in T regulatory cells and transforming growth factor-beta levels in response to treatment with vancomycin, indicating an immunomodulatory effect [119].

Although, according to a recent meta-analysis of previous RCTs, vancomycin might be the most effective antibacterial agent for PSC, a recent matched analysis in a pediatric population (n = 264) did not show any efficacy of oral vancomycin treatment (n = 88) compared to UDCA treatment (n = 88) or no treatment (n = 88) [115,120]. Moreover, despite the absence of major adverse events, antibiotic drug resistance is still an important concern. However, strikingly, a recent case series describing the use of oral vancomycin to treat colitis in 17 children suffering from PSC, did not reveal the presence of any vancomycin-resistant enterococcus in this small cohort [115,121]. At present, most available studies are limited by small sample size. Hence, large sample-sized RCTs with longer follow-up period and treatment duration are needed. Furthermore, the exact mechanisms of these microbiome-based therapies remain to be explored [112].

### 6.2. Fecal Microbiota Transplantation (FMT)

Fecal microbiota transplantation (FMT is the transfer of the fecal microbiota from a healthy individual to a patient in order to reverse gut dysbiosis and restore a normal balance in the lower gastrointestinal tract [122]. Several RCTs and case reports implicate that FMT has evolved as a promising treatment alternative for recurrent *Clostridioides difficile* infection and UC [123,124,125,126,127,128,129]. Allegretti et al. performed a pilot study with 10 patients with PSC and IBD in remission who received FMT from one donor. Transplanted OTU included genera capable of producing SCFA, which are known to be depleted in IBD [111]. Strikingly, 30% experienced a ≥50% decrease in ALP levels. Up to now, only one pilot study has been published, thus, larger sample-sized studies are needed to define efficacy and FMT mechanisms in PSC [111].

At present, most of the trials on the use of FMT have been stopped or at least revised, because two clinical studies in the USA reported bacteremia with Extended spectrum beta-lactamase-producing E. coli after transmission as part of FMT, resulting in the death of one patient [130].

## 7. Bile Microbiota in PSC

Bile is a biological fluid, consisting mainly of bile acids, cholesterol, phospholipids, and proteins. Another key component of human bile is bicarbonate, and impaired bicarbonate secretion is associated with liver damage. The concept of a biliary “bicarbonate umbrella” specifies that bile duct integrity depends on a protective bicarbonate layer that facilitates deprotonation of hydrophobic bile acids, thereby preventing cholangiocellular cytotoxicity [131,132]. Therefore, several treatment options in cholestatic liver diseases directly or indirectly stabilize the biliary HCO3-umbrella [132]. For instance, UDCA enhances the secretory capacity of hepatocytes and cholangiocytes, improving biliary HCO_3_-secretion [132].

Bile acids, which are the major organic solutes of human bile, are believed to play a crucial role in the pathogenesis of cholestatic liver diseases such as PSC. As conversion of primary bile acids into secondary, potentially noxious bile acids is thought to be primarily driven by the bacterial gut microbiome, microbial dysbiosis is expected to play a crucial role in PSC [23]. Moreover, even if bile acids do not represent the primary cause of cholestatic liver injury, it is believed that they may perpetuate disease progression by generating or maintaining a chemokine and cytokine response [133].

Emerging data using next-generation sequencing indicate that bile might not always be sterile, as assumed before, but can harbor a diverse bile microbiome [93,134,135,136]. Patients with PSC are characterized by ecological alterations of the ductal bile, including reduced biodiversity and expansion of pathogenic bacteria such as Enterococcus faecalis, which is associated with impaired intestinal permeability and mucosal inflammation due to its production of matrix metalloproteinases such as gelatinase [66,137]. Of note, microbial dysbiosis such as *Enterococcus* abundance is associated with an increase of the proinflammatory and potentially cancerogenic bile acid taurolithocholic acid [66]. Moreover, earlier culture-based studies suggested that biliary isolates of bacteria, such as *Enterococcus* faecalis, the most frequently identified genus in bile culture studies, and *Enterococcus gallinarum*, induce a T helper cell type 17 immune response in patients with PSC [56,138,139].

Currently, only a limited number of studies have investigated bile microbiota in PSC patients [66,93]. Peirara et al. detected only slight microbial alterations in patients with either biliary dysplasia or cholangiocarcinoma, while patients without disease complications showed virtually no microbial differences compared with controls. Nevertheless, their study results are limited by a small patient cohort. In contrast, after analysis of 46 PSC patients, Liwinski et al. suggested that biliary dysbiosis might be linked with elevated concentrations of the proinflammatory and potentially cancerogenic agent taurolithocholic acid [66]. They observed a significant increase of the facultative anaerobic phylum *Proteobacteria* in the bile fluid of patients with PSC. The increase of *Proteobacterica*, comprising many human pathogens, such as members of the Enterobacteriaceae family, is associated with increased epithelial oxygen availability, a hallmark of inflammation, epithelial dysfunction and disease [140]. In addition, bile fluid analysis of PSC patients revealed an increased frequency of known cholangitis pathogens such as *Enterococcus*, *Prevotella*, *Staphylococcus* and *Cutibacterium* [66]. Moreover, an increase in *Streptococcus* abundance was significantly associated with the number of ERC examinations and disease severity [93]. Furthermore, Liwinski et al. report an increased microbial burden in the bile with increased PSC duration.

Since there is only a limited number of studies investigating the association between bile microbiota and PSC, more research in this field is urgently needed. In particular, multi-center studies that include the impact of different environmental factors have to be performed in near future [66].

### 7.1. Bile Acid Pathways and Their Therapeutic Targets

Pharmacotherapies targeting bile acid pathways represent an active area of research for the treatment of PSC. A short overview of a selection of therapeutic agents is provided in Table 3.

UDCA is widely used. Several studies demonstrated a beneficial effect on liver biochemistries, but no long-term benefits such as liver transplant or death coule be shown up to now [141,142]. However, a study by Lindor et al. revealed an association between UDCA and an increased risk of serious adverse events when used at higher doses (28-30 mg/kg per day) [143]. Moreover, previous studies showed that the altered gut microbiota in PSC patients is independent of treatment with UDCA [27,59]. Further, Fickert et al. revealed that treatment with norursodeoxycholic acid (norUDCA), a derivative of UCDA, resulted in significant reductions of ALP levels and a similar safety profile (low incidence of pruritus) compared to the placebo group after 12 weeks of treatment duration [51,149]. Kowdley and colleagues conducted a RCT including 76 PSC patients to investigate obeticholic acid (OCA), a semi-synthetic FXR ligand. They demonstrated a significant decrease in ALP levels after 24 weeks treatment duration, however, a higher proportion of patients suffering from pruritus have been reported [145]. Trauner et al. investigated Cilofexor, a nonsteroidal FXR agonist, which led to lower ALP levels at week 12 regardless of UCDA treatment [51,146]. Bezafibrate, a ligand of the intranuclear receptor peroxisome-proliferator-activated receptor alpha (PPARα), is another therapeutic agent that showed evidence of an improvement in serum liver enzymes in RCT [147,148]. It leads to an increase in P-glycoprotein levels in the bile duct canaliculi, which in turn increases phospholipid levels in the bile, leading to protection of the bile from toxic bile acids (anti-inflammatory effect) [150]. Moreover, an association with superoxide dismutase expression in the liver has been reported (anti-oxidative effect) [151]. Furthermore, Lemoinne et al. performed a retrospective analysis on 20 patients with incomplete response to UDCA, revealing an improvement in ALP levels as well as in pruritus after addition of bezafibrate or fenofibrate, another PPARα agonist [152]. Another therapeutic agent is seladelpar, a selective peroxisome-proliferator-activated receptor delta (PPARδ) agonist that is the subject of an ongoing RCT (NCT04024813). Whether and how these therapeutic interventions interfere with the microbiota should be analyzed in follow-up studies.

## 8. PSC and CCA

### 8.1. PSC and CCA-Overview

CCA is the most common malignancy in patients with PSC and the sixth most common cause of cancer in the gastrointestinal tract in the western world world [153,154,155]. The prevalence of CCA in patients with PSC is between 7 to 13% [155]. Although the pathogenesis of CCA in PSC is largely unelucidated, inflammation-driven carcinogenesis concomitant with genetic and epigenetic alterations are thought to be the underlying factors [156,157,158]. However, the knowledge of predisposing risk factors is scarce and PSC-CCA development does not seem to be linked to duration and severity of PSC [159,160,161,162]. The leading hypothesis is that PSC causes multifocal strictures of the biliary tree, biliary infections, and accumulation of toxic bile acids, which results in an increased expression of adhesion and antigen-presenting molecules and inflammatory mediators. Macrophages trigger the apoptosis and senescence of cholangiocytes in activated T cells and the production of proinflammatory chemokines and cytokines acting on hepatic stellate cells leads to liver fibrosis. In response, cholangiocytes lining the biliary tree activate a wide range of cellular processes such as hepatocellular proliferation, apoptosis, angiogenesis and fibrosis as well as immunoregulatory functions, such as upregulation of human leukocyte antigen molecules and recruitment of immune cells. However, it remains unclear if activated cholangiocytes directly activate B cell antibody production and T cell expansion [162]. Moreover, the biliary epithelial injury-induced regenerative response by peribiliary glands is also believed to be a key process involved in inflammation-induced CCA carcinogenesis [163,164]. The cholangiocytes and associated progenitor cells lining the biliary tree can undergo DNA damage, metaplasia, and low-grade dysplasia. In case of persistent stimuli, the disease may progress to high-grade dysplasia and ultimately CCA [156,157,165,166,167]. Emerging evidence already demonstrated that cholangiocytes in explant livers from PSC-CCA patients show metaplasia and dysplasia more often than in patients with PSC without CCA [162,168,169]. Strikingly, the majority of CCA cases develop from a dominant stricture, which is defined as a stricture with a diameter < 1.5 mm in the common bile duct or <1.0 mm in the hepatic duct [155].

CCA is the most common malignancy in patients with PSC and the sixth most common cause of cancer in the gastrointestinal tract in the western world world [153,154,155]. The prevalence of CCA in patients with PSC is between 7 to 13% [155]. Although the pathogenesis of CCA in PSC is largely unelucidated, inflammation-driven carcinogenesis concomitant with genetic and epigenetic alterations are thought to be the underlying factors [156,157,158]. However, the knowledge of predisposing risk factors is scarce and PSC-CCA development does not seem to be linked to duration and severity of PSC [159,160,161,162]. The leading hypothesis is that PSC causes multifocal strictures of the biliary tree, biliary infections, and accumulation of toxic bile acids, which results in an increased expression of adhesion and antigen-presenting molecules and inflammatory mediators. Macrophages trigger the apoptosis and senescence of cholangiocytes in activated T cells and the production of proinflammatory chemokines and cytokines acting on hepatic stellate cells leads to liver fibrosis. In response, cholangiocytes lining the biliary tree activate a wide range of cellular processes such as hepatocellular proliferation, apoptosis, angiogenesis and fibrosis as well as immunoregulatory functions, such as upregulation of human leukocyte antigen molecules and recruitment of immune cells. However, it remains unclear if activated cholangiocytes directly activate B cell antibody production and T cell expansion [162]. Moreover, the biliary epithelial injury-induced regenerative response by peribiliary glands is also believed to be a key process involved in inflammation-induced CCA carcinogenesis [163,164]. The cholangiocytes and associated progenitor cells lining the biliary tree can undergo DNA damage, metaplasia, and low-grade dysplasia. In case of persistent stimuli, the disease may progress to high-grade dysplasia and ultimately CCA [156,157,165,166,167]. Emerging evidence already demonstrated that cholangiocytes in explant livers from PSC-CCA patients show metaplasia and dysplasia more often than in patients with PSC without CCA [162,168,169]. Strikingly, the majority of CCA cases develop from a dominant stricture, which is defined as a stricture with a diameter < 1.5 mm in the common bile duct or <1.0 mm in the hepatic duct [155].

### 8.2. Gut Microbiota in CCA

The role of biliary microbiota has scarcely been investigated in CCA. However, previous studies performing 16S RNA sequencing demonstrated a significant difference of biliary microbiota composition between CCA patients and controls [162]. Initially, some authors suggested that bile fluid dysbiosis could be linked to various diseases, including biliary lithiasis. They hypothesized that the reduction of the biliary flow by (partial) obstruction could cause a change in biliary microbiota composition [135,170,171,172]. Although bacteria may find a more favorable growth environment in obstructed bile, this likely does not represent the only reason for differential colonization. It has been indicated that altered bile metabolism may provide metabolic advantages to certain bacteria, which might explain tumor growth and outcomes through host immune response to CCA [112,162]. Jia et al. were the first to characterize intestinal microbiota, bile acids, and cytokines in patients with CCA by analysis of fecal microbiota in a series of intrahepatic CCA (iCCA). Their findings revealed that the α-diversities and β-diversities of iCCA were highest and that the abundance of four genera (*Lactobacillus, Actinomyces, Peptostreptococcaceae and Alloscardovia*) was increased in patients with iCCA compared to non-iCCA patients (HCC, healthy, liver cirrhosis) [173]. Based on previous findings that suggest a different pattern of bile acid concentration in biliary cancer (compared to patients with benign biliary diseases), Jia et al. quantified bile acids in serum and stool [173,174]. They found that total serum bile acids were higher in patients with CCA compared to healthy controls. Moreover, they demonstrated increased plasma-stool ratios of glycoursodeoxycholic acid and tauroursodeoxycholic acid in patients with iCCA as well as a positive correlation of tauroursodeoxycholic acid with *Lactobacillus* and *Alloscardovia*, proposing them as diagnostic markers [173].

Avilés-Jiménez et al. compared mucosal microbiota (tissue samples) of extrahepatic CCA (eCCA) to benign biliary tumors and showed an abundance of *Methylophilaceae, Fusobacterium, Prevotella, Actinomyces, Novosphingobium* and *Helicobacter pylori* (HP) in eCCA [175]. However, despite the leading role of HP in various cancers, the presence of HP and Actinomyces are assumed to be gastric/intestinal contamination since these findings could not be confirmed in similar follow-up studies [162,173].

According to a recent study by Saab et al. (*n* = 28 CCA patients, *n* = 47 controls), the most abundant genera were *Enterococcus*, *Streptococcus*, *Bacteroides*, *Klebsiella*, and *Pyramidobacter* in CCA’s biliary microbiota. Strikingly, *Bacteroides Geobacillus*, *Meiothermus*, and *Anoxybacillus* genera levels were significantly higher in CCA patients’ biliary microbiota compared to the control group. Up to now, no link between carcinogenesis, especially CCA, and *Geobacillus, Meiothermus*, and *Anoxybacillus* levels could be demonstrated. In contrast, several studies have already demonstrated associations between Bacteroides and colon cancer, cholethiasis through metabolomic changes and various autoimmune diseases, such as arthritis in transgenic rats HLA-B27 [176,177,178,179,180].

Current literature on microbiota and PSC-CCA is scarce due to the fact that the pathogenesis of transformation from PSC to PSC-CCA remains unelucidated. Therefore, future studies should focus on the transformation of inflammatory changes of the biliary tree to cancer. Based on the results of these studies, further analysis on gut microbiota in PSC-CCA should follow [162,181].

## 9. Gallbladder Carcinoma—Overview and Role of Genotoxins in Carcinogenesis

Gallbladder cancer is the most common cancer of the biliary system and associated with a very poor prognosis. The interplay of genetic predisposition, lifestyle factors and infections in gallbladder carcinogenesis remains obscure. Its incidence is relatively high in the western parts of South America and in the northern part of the Indian subcontinent, while being rather uncommon in Western countries [182]. Strikingly, in these regions, an epidemiological association between gall bladder cancer and chronic infection with the bacteria *Salmonella enterica Typhi/Paratyphi A* exists. The leading hypothesis is that in these patients, Salmonella resides in the gallbladder by forming biofilms on gallstones, which facilitates enhanced colonization, persistence in this organ and carriage into the duodenum [183,184,185,186]. Boccellato and colleagues developed a three-dimensional organoid model that recapitulates the infection dynamics in the healthy gallbladder epithelium. Infection with *Salmonella*, in particular the typhoid toxin CdtB subunit, induces DNA double-strand breaks. With their advanced organoid model, the authors were able to see the toxin and DNA damage spread to neighboring cells that were not infected with *Salmonella*. While infected cells underwent a toxin-independent cell cycle arrest, uninfected intoxicated cells were able to continue proliferating despite the DNA damage, providing a potential opportunity for malignant transformation [187].

There is emerging evidence that bacterial infections and bacterial products from *HP, E. coli, Chlamydia trachomatis* and others can cause DNA damage in host cells by secreting genotoxic proteins or through mechanisms involving host response to the infection [188,189,190,191,192,193]. In addition to producing genotoxins or causing DNA damage through increased production of reactive oxygen species, bacterial infections can also modify the DNA damage response, thereby interfering with mechanisms of repair [188]. Recent studies provided evidence that colibactin, a bacterial toxin that is expressed in *E. coli* and *Klebsiella pneumoniae*, might have an important impact on cancerogenesis by directly causing DNA damage [194,195]. Recently, a specific colibactin-driven genomic signature has been found using organoids [195]. Meyer and colleagues also found DNA breaks at a distinct sequence motif upon infection and confirmed their occurrence in human cancer [194]. Furthermore, short-term exposure to colibactin-producing *E. coli* was sufficient to promote colonic organoid transformation [196]. Thus, future studies involving the genotoxic effect of specific bacterial products such as colibactin and their association to biliary cancer are urgently needed. It will be important to investigate whether and to what extent direct genotoxic effects also contribute to PSC-associated cancers.

## 10. Conclusions

Several clinical studies, which analyzed microbial bacterial composition in fecal, mucosal and bile samples have demonstrated that the gut microbiome in patients with PSC and PSC and IBD is different from that in healthy controls and IBD patients only. Moreover, the frequent concomitance of PSC and IBD indicates a critical role of the gut-liver axis in disease pathogenesis. Experimental and clinical data suggest that a diverse set of microbial functions may be relevant, including endogenous molecules produced by the microbiota, bacterial processing of pharmacological agents or dietary compounds and specific bacterial molecules or metabolites driving the immune process. With regard to carcinogenesis, there is emerging evidence that bacterial products can directly promote epithelial injury and act as genotoxins.

Despite crucial progress in this field over the past years, a better understanding of the functional characterization of PSC, the gut microbiome, and related malignancies is needed. Therefore, future research should perform cross-sectional and longitudinal studies with larger patient cohorts to identify the role of the microbiota in disease progression.

## Figures and Tables

**Table 1:** Summary of studies analyzing fecal, mucosal, and bile samples of patients with PSC.

Reference	Sample Origin	No. of PSC Patients with and without IBD (*n*=)	Microbiology Assessment	Major Findings with Regard to the Gut Microbiome
Sabino et al., 2016 [59]	Stool	66	16sRNA gene sequencing	Significant over-representation of *Enterococcus, Fusobacterium,* and *Lactobacillus genera*
Kummen et al., 2017 [27]	Stool	85	16sRNA gene sequencing	Enrichment of the *Veillonella genus*
Bajer et al., 2017 [25]	Stool	43	16sRNA gene sequencing	*Rothia*, *Enterococcus*, *Streptococcus*, *Veillonella* were overrepresented in PSC regardless of concomitant IBD Decreased abundance of *Adlercreutzia equolifaciens* and *Prevotella copri*
Iwasawa et al., 2017 [60]	Stool	27	16sRNA gene sequencing	Abundance of *Enterococcus* (*E. faecalis* especially), *Streptococcus* (with prevalence of *Streptococcus parasanguinis*) and *Veillonella* species *Parabacteroides* overrepresented
Rühlemann et al., 2019 [12]	Stool	137	16sRNA gene sequencing	Increased abundance in eight genera *Veillonella, Streptococcus*, *Lactobacillus*, *Enterococcus*; *Proteobacteria*, *Lactobacillales (Bacilli)*, *Parabacteroides*, and *Bacteroides*
Lemoinne et al., 2020 [61]	Stool	49	16sRNA gene sequencing	Fungal dysbiosis: increased proportion of *Exophiala,* decreased proportion of *Saccharomyces cerevisiae* Bacterial dysbiosis: *Firmicutes decreased (except Veillonella increased)* and *Proteobacteria* increased
Kummen et al., 2021 [62]	Stool	136	metagenomic shotgun sequencing	Increased abundance of *Clostridiales* species Depletion of *Eubacterium*, *Ruminococcus obeum*
Rossen et al., 2015 [63]	Ileum mucosa	12	16sRNA gene sequencing	Abundance of uncultured *Clostridiales II*
Kevans et al., 2016 [26]	Colon mucosa	31	16sRNA gene sequencing	No changes
Torres et al., 2016 [64]	Ileum, colon mucosa	20	16sRNA gene sequencing	significant increase in *Barnesiellaceae* and *Blautia*
Quraishi et al., 2017 [24]	Colon mucosa	11	16sRNA gene sequencing	significant increase in *Escherichia*, *Megasphera* and *Lachnospiraceae*
Quraishi et al., 2020 [65]	Sigmoid mucosa	20	16sRNA gene sequencing	significantly higher abundance of the genera *Bacteroides fragilis*, *Roseburia*, *Shewanella*, and *Clostridium ramosum* in patients with PSC-IBD compared to patients with UC
Liwinski et al., 2020 [66]	Duodenal and oral fluids, duodenal mucosa, bile	46	16sRNA gene sequencing	Duodenal mucosa biopsy: overrepresentation of *Escherichia coli* and *Veillonella dispar* Bile fluid: overrepresentation of *Enterococcus* (especially *Enterococcus faecalis*), *Proteobacteria Staphylococcus*, *Neisseria*, *Veillonella dispar*

PSC, primary sclerosing cholangitis; IBD, inflammatory bowel disease; UC, ulcerative colitis.

**Table 2:** Overview of therapeutic agents targeting gut microbiota and their study characteristics.

References	Therapeutic Agents	Study Type	PSC Patients (N=)	Treatment Duration	Primary Endpoint	Results
Rahimpour et al., 2016 [106]	Vancomycin	RCT	8	12 weeks	Decrease in MRS at week 12	Significant reduction in mean MRS and ALP levels
Tabibian et al., 2013 [107]	Vancomycin vs.Metronidazole	RCT	9 9	12 weeks	Decrease in ALP levels at week 12	Non-dose dependent significant ALP reduction (for low and high dose vancomycin)
Färkkilä et al., 2004 [108]	Metronidazole (and UDCA)	RCT	39	36 months	Decrease in ALP or other liver enzymes, MRS, symptoms or histology at week 36	Significant ALP level and MRS reduction, improvement (of stage and grade) in histology
Tabibian et al., 2017 [109]	Rifaximin	Open-label study	16	12 weeks	Decrease in ALP levels at months 3	No significant reduction
Silveira et al., 2009 [110]	Minocycline	Open-label study	16	1 year	Decrease in ALP levels at month 12	Significant ALP reduction and mean MRS
Allegretti et al., 2019 [111]	Fecal transplantation	Open-label pilot	10	24 weeks	≥50% ALP reduction at week 24	Significant reduction in ALP levels in 3/10

Mayo risk score (MRS); number of treated PSC patients (N); ursodeoxycholic acid (UDCA); randomized controlled trial (RCT); alkaline phosphatase (ALP).

**Table 3:** Overview of a selection of therapeutic agents targeting bile acid pathways and their study characteristics.

References	Therapeutic Agents	Target Receptor/ Mechanism	Study Type	N	Treatment Duration	Primary Endpoint	Results
Beuers et al., 1992 [141]	UDCA 13–15 mg/kgper day	Choleretic effect, unknown receptor	RCT	5	12 months		Significant ALP reduction
Olsson et al., 2005 [142]	UDCA 17–23 mg/kgper day	Choleretic effect, unknown receptor	RCT	110	5 years	Occurrence of liver transplantation	Significant improvement in ALP levels, no impact on survival
Lindor et al., 2009 [143]	UDCA 28–30 mg/kgper day	Choleretic effect, unknown receptor	RCT	76	6 years	Changes in ALP and other liver enzymes	Significant improvement in ALP levels, no impact on survival, higher doses are associated with a higher rate of serious adverse events
Fickert et al., 2017 [144]	NorUDCA	Choleretic effect, unknown receptor	RCT	161	12 weeks	Mean relative change in ALP levels	Significant dose-dependent ALP reductions
Kowdley et al., 2020 [145]	OCA	Endogenous FXR agonist	RCT	76	24 weeks	Change in ALP at week 24	Significant reduction in ALP levels (only for use of OCA 5–10 mg)
Trauner et al., 2019 [146]	Cilofexor	Nonsteroidal FXR agonist	RCT	42	12 weeks	Change in ALP and other liver enzymes at week 12	Significant reduction in ALP levels and other liver enzymes
Mizuno et al., 2010 [147]	Bezafibrate	PPARa-agonist	Open-label pilot	7	6 months	Change in ALP levels at month 6	Significant ALP reduction
Mizuno et al., 2015 [148]	Bezafibrate	PPARa-agonist	Open-label pilot	11	12 weeks	Improvement in liver function tests	Significant ALP reduction
NCT04024813	Seladelpar	Selective PPARδ agonist	RCT		24 weeks	Change in ALP at week 24	Ongoing

Ursodeoxycholic acid (UDCA); norursodeoxycholic acid (norUDCA); obeticholic acid (OCA); farnesoid X receptor (FXR); number of treated PSC patients (N); peroxisome-proliferator-activated receptor delta (PPARδ); peroxisome-proliferator-activated receptor alpha (PPARα).

## Data Availability

Not applicable.

## Abbreviations

Alkaline phosphatase	ALP
Cholangiocarcinoma	CCA
Crohn’s disease	CD
Endoscopic retrograde cholangiopancreatography	ERCP
Extrahepatic CCA	eCCA
Fecal microbiota transplantation	FMT
Free fatty acids	FFA
Helicobacter pylori	HP
Inflammatory bowel disease	IBD
Intrahepatic CCA	iCCA
Lipopolysaccharide-binding protein	LBP
Pathogen-associated molecular patterns	PAMPs
Peroxisome-proliferator-activated receptor alpha	PPARα
Peroxisome-proliferator-activated receptor delta	PPARδ
Multidrug resistance 2	mdr2
Mucosal addressin cell-adhesion molecule 1	MAdCAM1
Mycophenolate mofetil	MMF
Nonalcoholic fatty liver disease	NAFLD
Norursodeoxycholic acid	norUDCA
Operational Taxonomic Units	OTU
Primary sclerosing cholangitis	PSC
Short-chain fatty acids	SCFA
Ulcerative colitis	UC
Ursodeoxycholic acid	UDCA
Vascular adhesion protein-1	VAP-1
